# The challenges of long-term follow-up data collection in non-commercial, academically-led breast cancer clinical trials: the UK perspective

**DOI:** 10.1186/1745-6215-15-379

**Published:** 2014-09-27

**Authors:** Lucy S Kilburn, Jane Banerji, Judith M Bliss, on behalf of the NCRI Breast Clinical Studies Group

**Affiliations:** ICR Clinical Trials & Statistics Unit (ICR-CTSU), Division of Clinical Studies, The Institute of Cancer Research, Sir Richard Doll Building, Cotswold Road, London, SM2 5NG UK

**Keywords:** Breast cancer, Long term follow-up, Randomised controlled trials, Data collection

## Abstract

**Background:**

Improved survival rates in early breast cancer and the chronic nature of disease relapse result in a large cohort of patients being available for long-term follow-up (LTFUP) in randomised controlled trials. Whilst of recognised scientific value to assess long-term treatment-related sequelae, the volume of this activity can be challenging for trialists and participating sites, and comes at a considerable cost to research funders and the National Health Service (NHS). A National Cancer Research Institute Breast Clinical Studies Group supported project aimed to characterise UK LTFUP data collection procedures in order to propose improvements.

**Methods:**

Protocols and case report forms for UK non-commercial National Institute for Health Research portfolio early breast cancer randomised controlled trials were reviewed and a questionnaire sent to associated participating NHS sites. Responders were asked to give opinions on issues with follow-up and LTFUP data collection procedures and to suggest potential improvements to practice. Results were used to inform design of a proposed standard LTFUP case report form.

**Results:**

Thirty-four trials, involving eight Clinical Trials Units were eligible for inclusion in the review. All trials requested follow-up at least annually up to 5 years, with two-thirds requesting LTFUP after that time point. Information relating to efficacy endpoints was captured for all trials via case report forms; however, precise detail on recording of recurrence, second malignancies and death varied. Separately, questionnaires were returned from 66 NHS sites. Main concerns identified included difficulties in identifying all adverse events from hospital notes, volume of work, bureaucratic data management practices in Clinical Trials Units and perceptions of prioritisation of recruitment over follow-up.

**Conclusion:**

Variation has existed with respect to detail of LTFUP information requested for UK trials. Improved communication, simplification and standardisation of data and associated collection methods are possible without compromising data requirements for efficient and effective trial reporting. Future use of routinely collected data, captured via electronic means, could transform practices and alleviate resource usage.

**Electronic supplementary material:**

The online version of this article (doi:10.1186/1745-6215-15-379) contains supplementary material, which is available to authorized users.

## Background

A consequence of the welcome improvement in the survival rates from selected cancers [1] is the increase in the number of patients available for long-term follow-up (LTFUP) in randomised controlled trials (RCTs), thus enabling further evaluation of long-term treatment-related sequelae beyond the time of primary analysis. Collation of existing evidence from historical trials in the form of meta-analysis (for example, by the Early Breast Cancer Trialists’ Collaborative Group) has illustrated that clinically important effects may only become apparent 10 or even 20 years after treatment was delivered. In particular, trials investigating the addition of radiotherapy have demonstrated clinically worthwhile effects on long-term outcome from breast cancer; however, they have also identified an apparent excess risk of non-breast cancer deaths, most notably from cardiovascular disease. In both cases, divergence between the treatment groups did not begin to emerge until many years after treatment [2]. In terms of systemic therapy for breast cancer, the full potential of tamoxifen was not in fact observed until 10 years after randomisation [3]. Conversely, possible carcinogenic risks associated with cytotoxic therapy for breast cancer may not be expected to be seen for many years after treatment. Therefore, whilst an analysis at or around 5 years follow-up may be reasonable to provide an initial estimate of treatment effect on disease-free survival, lack of LTFUP beyond that point misses the opportunity to characterise whether such beneficial effects of treatment translate into improvements in overall survival, and also incurs the risk of failing to detect potential longer term safety issues.

Given the large number of patients worldwide prescribed cancer treatment on the basis of trial evidence, trialists including those conducting breast cancer trials have a responsibility to ensure that trials report full and unbiased evidence on both the safety and efficacy of trial treatments. This requires follow-up of trial patients for many years after they have completed their treatment which inevitably incurs work for both sites recruiting patients into trials and Clinical Trials Units (CTUs) collecting and analysing the data.

Phase III trials in early breast cancer typically recruit 3,000 to 4,000 patients and utilise disease-free survival as their primary endpoint (a composite of local, distant relapse and death) analysed via survival analysis methods at a follow-up of about 5 years. With recruitment likely to be ongoing for 3 to 5 years, a decade can often elapse between the time a funding application is submitted and the time point when the majority of patients become eligible for LTFUP. Considerable changes can have occurred in expectations and practices of funders and health services during that time period and the proportion of patients surviving and available for LTFUP may be more than originally expected due to concurrent general improvements in patient care and associated outcomes.

Thus, the issues associated with the current follow-up and in particular LTFUP activity of trial patients are twofold. First, the number of UK patients entered into trials who continue in follow-up is now extensive. For example, over 13,000 UK patients were recruited to the National Epirubicin Adjuvant Trial (NEAT), Adjuvant Breast Cancer (ABC), Taxotere as Adjuvant Chemotherapy (TACT), Intergroup Exemestane Study (IES), and Standardisation of Breast Radiotherapy (START) breast cancer trials, and the approximate percentages of participants surviving at 5 years after randomisation for these trials are 79%, 78%, 82%, 90%, and 89%, respectively. Thus, completion of follow-up and LTFUP case report forms (CRFs) is a considerable resource activity for research staff in participating sites. Similarly, the CTUs continue to employ data management staff to oversee receipt of follow-up and LTFUP data, requiring funding for a dedicated resource lasting for many years after a trial has reported its principal results. Historically this work has been largely unquantified. As the volume of work has increased, however, given the growth in the number of trials being undertaken and the improved survival rates observed, together with the fact that oversight of trial activities in both CTUs and participating sites is more heavily scrutinised, a concern has grown as to the potential burden of associated work leading to the need to quantify the current activity and to ensure procedures are efficient and appropriate to justify the continued resource dedicated to this activity. Second, as National Health Service (NHS) clinical practice changes, more patients are being discharged earlier from oncology care. Hence, the ability of participating sites to refer to routine data sources (for example, hospital records) in order to obtain the requisite follow-up information is increasingly threatened and requires participating sites to chase individual patients via General Practitioners (GPs) or contact the patients themselves, potentially causing unnecessary distress.

In the UK, clinical trial investigators and CTUs have been aware of the opportunities and challenges associated with follow-up and in particular LTFUP for many years and advocate minimising data collection requirements to ease some of the burden on participating sites. One such way trial teams are trying to reducing this burden is by collecting patient identifiers at trial entry, with patient consent, to enable use of tracing systems and with a desire in future to link with routinely available datasets - for example, cancer registry data. However, each trial also traditionally has bespoke CRFs with format, data collection items and the frequency of follow-up having the potential to vary between trials. Anecdotal evidence suggested some trials, both newer and older, were requesting surplus information or data that is almost impossible to retrieve within sites and at too frequent intervals. In addition, it has been recognised that the balance between minimising losses to follow-up and timely collection of important clinical information needs to be identified and consideration given to the actual information requested and its impact. For example, standardised recording of normal tissue damage after radiotherapy is not routinely available in oncology notes but is essential for evaluation of normal tissue cosmetic outcomes; therefore, the additional resource required to collect these data manually can be justified. Alternatively, collecting data on hospitalisations for patients during LTFUP may not be cost-effective, especially as these events are unlikely to be associated with treatment sequelae, are likely to occur in hospitals other than the follow-up site and, as a consequence, result in data of questionable completeness and validity.

Here, the results of a project, funded by the National Institute for Health Research (NIHR) National Cancer Research Network (NCRN) and National Cancer Research Institute (NCRI) and conducted on behalf of the NCRI Breast Clinical Studies Group are reported. The aims of the project were to characterise the type and quantify the volume of LTFUP activity in non-commercial, academic-led RCTs in early breast cancer. The project included a protocol and CRF review to determine current practices in RCT data collection and a questionnaire to NHS participating sites.

## Methods

### Protocol and case report form review

Trials eligible for the protocol and CRF review were those early breast cancer RCTs which were listed on the NIHR portfolio in 2008 (Table 1). Trials were included if they were designed to investigate the effects of neoadjuvant or adjuvant therapy, systemic therapy or radiotherapy and where recurrence (as assessed via disease-free survival or similar endpoint) or survival was a primary or secondary endpoint. Such trials would routinely be expected to collect data over many years and to continue to quantify disease outcomes and risks beyond the time of the analysis of primary endpoint.

**Table 1:** **List of all trials present on the National Institute for Health Research trials portfolio in 2008 eligible for the review of Clinical Trials Unit current practice**

• ACTION	• PERSEPHONE
• ALTTO	• POETIC
• ATTOM	• PRIME
• AZURE	• PRIME II
• COMICE	• REACT
• DEVA	• SECRAB
• EORTC 10981	• SOFT
• ESTEEM	• START
• FAST	• SUPREMO
• HERA	• TACT
• HRT	• TACT2
• IMPORT HIGH	• TANGO
• IMPORT LOW	• TEAM
• NEAT	• TEXT
• NEO EXCEL	• TOPIC
• NEO TANGO	• TOPIC2
• NEOCENT	• TRAFIC

For each trial identified, the relevant CTU responsible for central management of the trial and Chief Investigator were contacted and asked to send trial protocols and CRFs for review. Data was extracted for each trial to characterise the trial’s follow-up and LTFUP data requirements. Data extraction was conducted using a five-page proforma which aimed to extract relevant information such as frequency of data collection, patient identifiable data collected at baseline and efficacy and adverse event (AE) question definitions (Additional file 1: Figure A1). Statistical analysis was descriptive in nature, and summarised in tabular and graphical form. Unless otherwise stated, all information was taken from the (repeating) follow-up form only (for example, recurrence information requested on follow-up form only, regardless of whether information is requested again on a separate one-off recurrence form).

### Questionnaire to National Health Service participating sites

Subsequent to the protocol and CRF review, a decision was made to develop a questionnaire to send to NHS participating sites to ascertain a better understanding of the issues relating to LTFUP in breast cancer trials from a site perspective.

In November 2009, a questionnaire was distributed via the NCRN and their research network managers to NHS participating sites. This method of distribution aimed to ensure a co-ordinated approach and optimise return rates (Additional file 1: Figure A2); however, this results in an unknown denominator in terms of number of sites surveyed. Given that the project was a survey of NHS staff in their professional role, ethical approval was not required. The survey requested that the questionnaire be completed in relation to experiences of non-commercial, academic-led breast cancer clinical trials on the NIHR portfolio only, by any person responsible for completing follow-up forms associated with such trials. A general reminder email was sent to encourage sites to return questionnaires. Responses were assessed on a site-by-site basis. The questionnaire asked about routine practice in sites and accessibility of data items currently requested for breast cancer trials. Responders also had the opportunity to give opinions on issues with LTFUP data collection in their participating site, issues with electronic follow-up and to propose any solutions they considered helpful. Qualitative free text answers were assessed for key themes.

### Definition of long-term follow-up

There is no standard definition for “follow-up” or “LTFUP” in RCTs. Therefore, for this project “follow-up” was defined as visits/contact subsequent to completion of surgery/radiotherapy/chemotherapy treatment and “LTFUP” as visits/contact >5 years after randomisation. Follow-up at participating sites is normally conducted via patients attending an outpatient appointment or staff at participating sites, a request to the patient’s GP or by contacting the patient by telephone.

### Quantifying the volume of activity associated with long-term follow-up

To represent the volume of activity in the UK of following up breast cancer trial patients >5 years after randomisation, the number of patients previously entered into NIHR portfolio RCTs and continuing to be followed-up was estimated by year. This was calculated using annual recruitment rates in the UK up to the end of 2009 for trials eligible for protocol and CRF review (see below) and 5-year survival rates for these trials if available (or an approximation if not). It also assumed an approximate lost to follow-up/death rate of 4% for year 6 onwards.

Results of the data extraction from protocols and CRFs and quantitative responses in the questionnaires returned from participating sites were collated via entry into an Access database (Microsoft Corporation, Redmond, WA. USA). All statistical analyses were conducted using Stata version 10 (StataCorp, College Station, Texas, USA).

## Results

**Figure 1:**
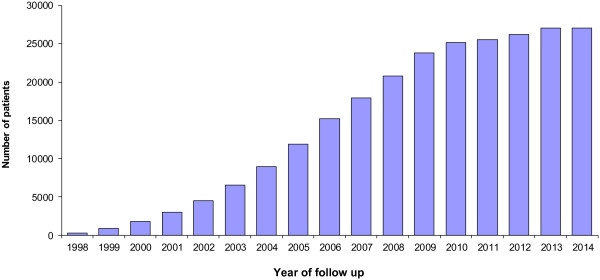
**Growth in UK patients predicted to be in long-term follow-up (>5 years after randomisation).** Thirty-four national trials were included in the review. It is expected that approximately 27,000 patients would be in long-term follow-up by the end of 2013.

Thirty-four trials were deemed eligible to be included in the protocol and CRF review. Whilst these trials represent a cross-section of NIHR portfolio trials it can be assumed that, for trials included in the review, the number of patients in LTFUP would have reached steady state in 2013 when approximately 27,000 patients in the UK would have been >5 years after randomisation and still available for follow-up (Figure 1). This number would be expected to decline gradually over the next few years due to a small number of patients dying or becoming unavailable for follow-up each year.

### Protocol and case report form review

**Figure 2:**
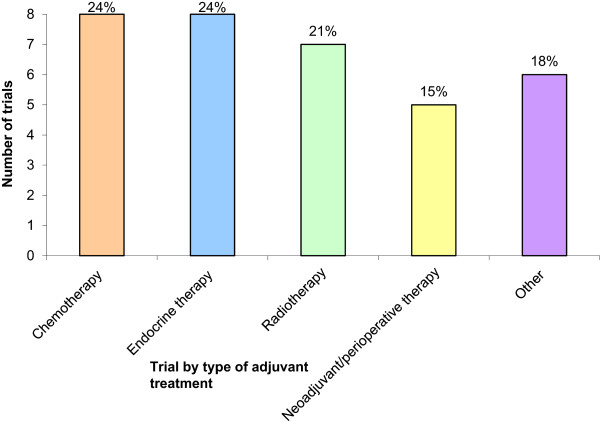
**National trials included in the review.** Data on the type of adjuvant treatment was taken from either the protocol or case report form.

Of the 34 eligible trials, only two trials declined to provide CRFs for the project and information for these trials was obtained from the protocol only. Standard adjuvant therapy trials evaluating chemotherapy, endocrine therapy and/or radiotherapy comprised nearly two-thirds of the trials in the review, with the minority remainder including trials of targeted therapies and neoadjuvant/perioperative treatment - the more recent focus of the NIHR breast cancer portfolio (Figure 2).

#### Patient identifiers and tracing patients long-term

Nearly all of the trials collected date of birth (32/34 (94.1%)) and hospital number (29/34 (85.3%)) with just under three-quarters collecting the full patient name (25/34 (73.5%)). NHS number, the key data item used for identifying patients in registry data, was only collected in approximately half of the trials (18/34 (52.9%)) (Additional file 1: Figures A3a,b). As a consequence of collecting patient identifiers, 21 (64%) trials stated they are intending to use tracing services to facilitate follow-up of patients in the long term.

#### Timing of follow-up

Nearly all trials aim to collect information on the anniversary of randomisation at years 1 and 2 (year 1 = 32/34 (94.1%); year 2 = 33/34 (97.1%)), with half of the trials requesting intermediate follow-up data at 18 months (17/34 (50.0%)). A similar pattern is present between years 2 and 5, with all trials collecting data annually (from year 2 = 33/34 (97.1%); from years 3 to 5 = 34/34 (100.0%)) and a few trials collecting data in between at 2.5 and 4.5 years (2.5 years = 14/34 (41.2%); 4.5 years = 8/34 (23.5%)). Annual data collection continues between years 5 and 10. Approximately two-thirds of trials (22/34 (64.7%)) state an intention to collect data routinely beyond 5 years (Additional file 1: Figures A4a-c).

#### Efficacy data

All trials collected some information on efficacy data on the follow-up form. The patient’s vital status was collected in 31 (91.2%) trials, while the date the patient was last seen or contacted was collected for 32 (94.1%) trials; 26 (76.5%) trials requested that the patient was seen in person, 3 (8.8%) allowed the patient to be seen or contacted and 3 (8.8%) did not specify. Disease outcome status, including date of death, local/distant recurrence and second primary details, were requested on 21 (61.8%), 32 (94.1%) and 24 (70.6%) of follow-up forms, respectively, with some requesting an additional recurrence/death form be completed as appropriate. Other trials took a reactive approach and required completion of separate recurrence/death forms only when an event occurred. Five (14.7%) trials requested death certificates or autopsy reports from participating sites and 3 (8.8%) routinely collected pathology and radiology reports for recurrence or second primary events. In addition, half the trials (17/34) collected details on treatment after relapse.

#### Safety information on follow-up form

The collection of data relating to safety and occurrence of AEs was varied (Table 2) with data requested on specific types of AEs via tick boxes, often using Common Toxicity Criteria for Adverse Events, or Radiation Therapy Oncology Group grading, by the type of investigational treatment. Endocrine therapy trials most commonly collected information on cardiovascular, musculoskeletal and gynaecological events. Radiotherapy trials collected cardiovascular events and fractures. All trials allowed a free-text space to provide information on “other” toxicity. Seven trials collected data relating to patient hospitalisations during LTFUP as a free-text option; three collected hospital admissions only while the remaining four also requested details on specific AEs.

**Table 2:** **Number of trials recording adverse events by type of treatment**

	Chemotherapy	Endocrine therapy	Radiotherapy	Neoadjuvant/perioperative therapy	Other	All
Cardiovascular	3 (38%)	4 (50%)*	5 (71%)*	0 (0%)	2 (33%)	14 (41%)
Neurological	1 (13%)	2 (25%)	0 (0%)	0 (0%)	2 (33%)	5 (15%)
Menopausal	0 (0%)	3 (38%)	0 (0%)	0 (0%)	0 (0%)	3 (9%)
Fracture	1 (13%)	4 (50%)*	5 (71%)*	0 (0%)	1 (17%)	11 (32%)
Other musculoskeletal	1 (13%)	4 (50%)*	2 (29%)	0 (0%)	0 (0%)	7 (21%)
Psychiatric	0 (0%)	2 (25%)	0 (0%)	0 (0%)	0 (0%)	2 (6%)
Gastrointestinal	0 (0%)	3 (38%)	0 (0%)	0 (0%)	0 (0%)	3 (9%)
Gynaecological	0 (0%)	4 (50%)*	0 (0%)	0 (0%)	0 (0%)	4 (12%)
Other (free text)	6 (75%)*	6 (75%)*	5 (71%)*	3 (60%)*	4 (67%)*	24 (71%)*
Hospitalisations (free text)	3 (38%)	2 (25%)	0 (0%)	0 (0%)	2 (33%)	7 (21%)

*Greater than or equal to 50% of trials requested details.

### Questionnaire to National Health Service participating sites

Questionnaires were returned from 66 UK NHS participating sites. The majority of the questionnaires were completed by research nurses and data managers responsible for completing trial CRFs at the participating site. The median number of academic-led early breast cancer (EBC) RCTs running per site was 11.5 (interquartile range: 8 to 14). Clinics run specifically to follow-up trial patients were available in 13/66 (19.7%) sites. Of those sites that did not have specific follow-up clinics, 17 (32.1%) have considered setting one up, with the main reason for not having set up one being lack of space/time (n = 9) and awaiting decision/outcome of trial (n = 5). Of those sites that have specific follow-up clinics, 69% have them on a weekly basis.

**Figure 3:**
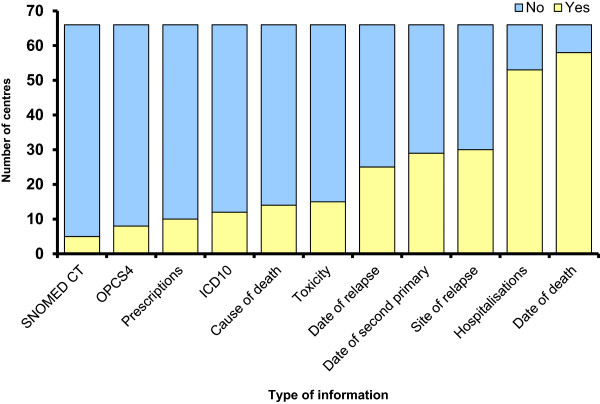
**Information available on hospital electronic patient record systems.** Data were obtained from returned questionnaires (see Additional file 1: Figure A1). ICD10, International Classification of Diseases-10; OPCS4, Operating Procedure Codes-4; SNOMED CT, Systematized Nomenclature of Medicine Clinical Terms.

Responders also indicated what information was available on their local electronic patient record systems (Figure 3). Access to clinical coding systems (for example, Systematized Nomenclature of Medicine Clinical Terms, International Classification of Diseases-10, Operating Procedure Codes-4) was limited, and approximately half the responders had electronic access to relapse and second primary cancer information and date of death easily available, but cause of death was more difficult to ascertain. In addition, the majority of responders stated that hospital admissions and toxicity data were only accessible if they were reported in the same NHS Trust. AEs were not reported systematically either electronically or on paper records.

Responders’ views on LTFUP highlighted a number of common themes. Main concerns included difficulties in identifying all AEs, volume of work, bureaucratic data management practices in CTUs and perceptions of prioritisation of recruitment over follow-up (see below).

#### General issues with long-term follow-up collection in breast cancer trials – key themes

-  Lack of time/follow-up patient visits rescheduled
-  Lack of personnel/staffing issues (for example, continuity of staff)
-  Lack of tracking database/electronic systems
-  GPs uncooperative
-  Lack of space to store CRFs/archiving
-  (Early) discharge of patients
-  Transfer of patients to another hospital/lack of integrated electronic patient records system in UK
-  Information detailed in patient notes inadequate
-  Volume of patients in clinics
-  CTUs sending large batches of requests at one time
-  CTUs sending requests for really old data
-  CTUs expecting a short turnaround for requests
-  Lack of response from other departments within same hospital (for example, histopathology)
-  CTUs creating complex CRFs with unnecessary information/too many pages
-  CTUs creating new (additional) CRFs (for example, TACT Herceptin form)
-  Patients not turning up for follow-up visits (for example, not interested, feeling well or parking/travel costs)
-  Research and development departments and consultants not taking into account work required for follow-up when agreeing to take part in new trials
-  CTUs not talking to one another when sending out large requests
-  NCRN priorities e.g. recruitment over follow-up
-  CTUs sending out duplicate requests for data
-  Differing follow-up schedules between trials and with routine practice
-  CTUs requiring the follow-up date is brought back in line with date of randomisation
-  Difficulty (and/or cost) of retrieving patient notes (for example, from secure off-site storage facility)
-  Consultants telling patients there is no need to follow-up anymore, but then the trial insists on long term follow-up and therefore, the patient gets worried
-  Postage/photocopying/telephone costs

#### Issues with electronic follow-up collection – key themes

-  Passwords – remembering them, losing them, staff going off sick and so forth
-  Varying software/database quality
-  Lack of training
-  Firewalls
-  Internet connectivity/network issues
-  Electronic CRFs designed badly/too restrictive (for example, too many checks/not allowing missing data)
-  Not accessible in clinic
-  Lack of paper trail/back-ups
-  Lack of computers/access to internet/space for personal computers
-  Lack of IT support
-  Security/data protection/quality assurance/information governance issues
-  Electronic CRFs are time consuming to complete
-  UK compatability (for example, Scotland versus England)

### Standard long-term follow-up case report forms

**Figure 4:**
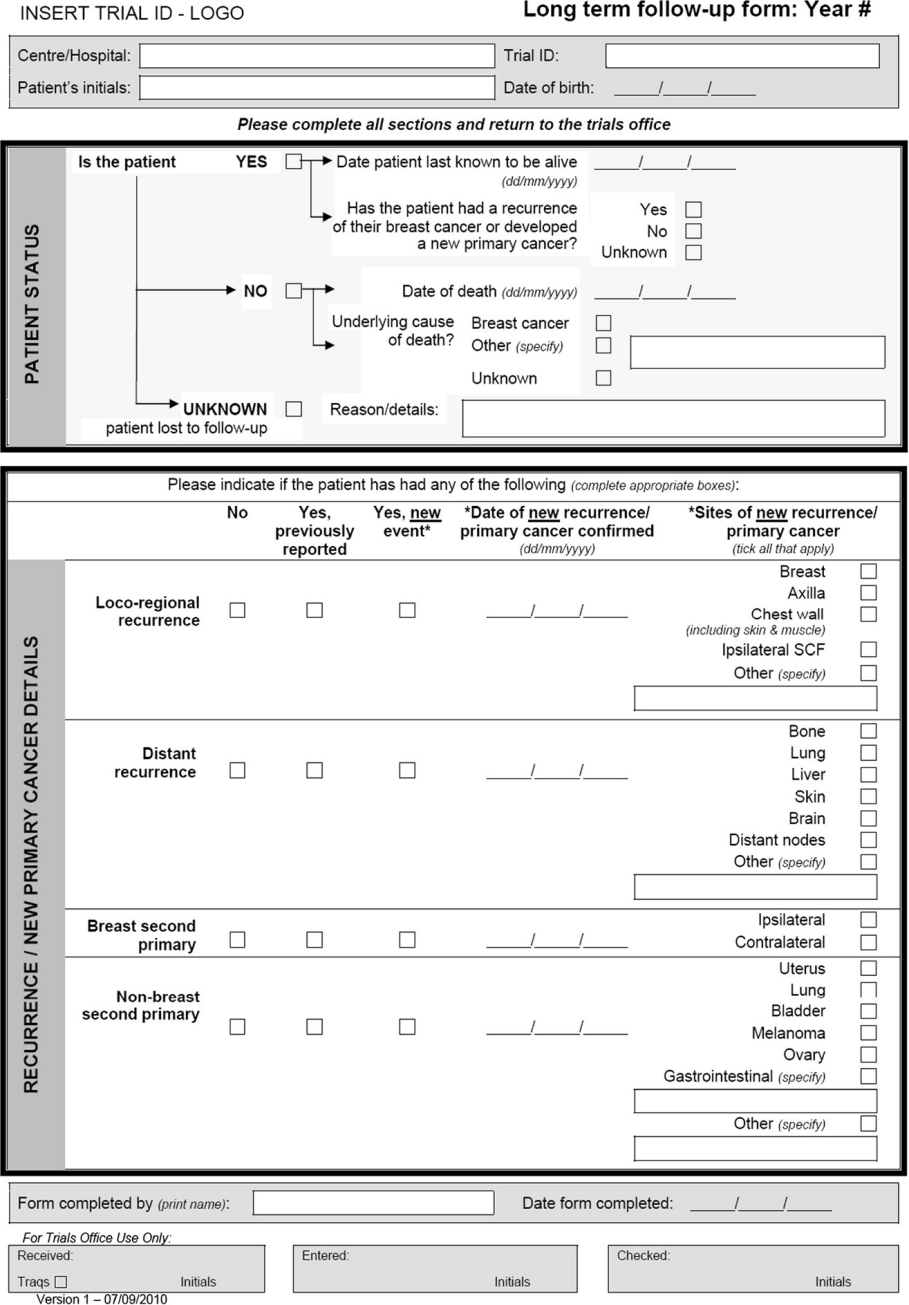
**Long-term follow-up form.** Simple one page CRF for collection of primary and secondary efficacy endpoints.

Feedback from the protocol and CRF review and site questionnaire contributed to the development of a standard LTFUP CRF that was endorsed by the NCRI Breast Cancer Study Group in September 2010 for use in current and future breast cancer trials to alleviate immediate challenges (Figure 4). The form was designed to be completed annually around the anniversary of patient entry into the trial (at randomisation) but with an unrestricted time window. It was supplied as a word document for electronic completion or it could be printed out and, given the data items included on the form, it was intended that there would be no need for additional relapse/death forms. Guidance notes accompanied the form to aid completion and maximise consistency of reporting. The form allowed the option for patients to be also contacted by telephone subject to approval within the trial and participating NHS Trust.

Feedback obtained approximately 1.5 years after endorsement of the CRF indicated that three trials that were part of the review have either adopted the form as it was created or produced a modified version based on its template; three CTUs stated that had not discussed using it for the specific trials in question but would consider using the form in future in the absence of an alternative to site-based data collection.

## Discussion

In breast cancer trials, the volume of activity associated with follow-up and subsequently LTFUP data collection is a significant and immediate challenge to trialists and to NHS participating sites, yet with breast cancer patients remaining at risk of relapse and subsequent death from their disease 15 years after diagnosis, the clinical requirement to collect this information is evident [2–5]. The protocol and CRF review conducted within this project showed that the frequency of LTFUP data collection has been consistent across trials, although earlier follow-up time points appear more varied and for some trials perhaps too unnecessarily frequent. Few trials have collected LTFUP data more frequently than annually and a new trial would need to justify an increase to this frequency given the resource implication. The review together with the questionnaire to sites also confirmed that some data items can be collected more easily than others; for example, as may be expected, recurrence and death details were more accessible than incidence and severity of AEs. Long-term side effects appear to be the most difficult to collect efficiently and systematically, yet are extremely important to help our overall understanding of these interventions.

Variation exists with respect to detail of LTFUP information collected across trials, influenced by the coordinating CTU. This has resource and funding implications for both participating hospitals and CTUs. Many clinical trials recruit UK-wide, and for some trials access to registry data is already requested although this has historically required access to several routine data sources given there has not been a single point of access for such data. In addition, Cancer Registries have been known to have a significant lag-time for receiving data. Historically, this has been approximately 18 months from the event occurring, while for a clinical trial with site-based follow-up the lag-time is approximately 6 months (on average) for LTFUP data. While these issues are being resolved, CTUs are encouraged to standardise data collection across trials by adopting the use of a standard CRF. The CRF developed as part of this project was designed to be simple and flexible whilst efficiently collecting the data required for the primary and secondary efficacy endpoints.

Whatever the method used to collect LTFUP data, it is necessary to be able to correctly identify patients and obtain appropriate permissions to follow their progress through routine data sources. The unique NHS/Community Health Index (CHI) number provides the ability to track patients throughout the UK, and thus is used as the primary identifier in registry datasets. Despite this we found only half of the trials in the review were collecting NHS/CHI numbers. We urge all investigators to collect NHS/CHI numbers routinely in clinical trials and to ask for patients’ consent to use routine data sources for LTFUP at the time of trial entry.

Given the mode of distribution of the LTFUP questionnaire to participating sites, via NCRN and its Research Network Manager network, it is not possible to easily determine the observed response rate. What is known, however, is the typical number of NHS sites participating in the large adjuvant breast cancer trials which is usually in the region of 100 to 120 NHS sites. Returned questionnaires from 66 sites would thus indicate a response rate in the region of 50%. This would imply a broad range of sites responded to the survey and, therefore, it is reasonable to assume that the main concerns all sites participating in EBC RCTs have with LTFUP have been identified. Feedback from participating sites via the LTFUP questionnaire identified common concerns, with workload and communication with CTUs being the main issues.

The ongoing necessity to ascertain information in the long term relating to patients entered into large cancer trials requires researchers to explore alternative methods for data collection. One such method, likely to be of increased utility given improvements in technology, is to seek to access routinely collected data to improve reliability of, for example, hospitalisations for long term AEs. The National Cancer Data Repository (NCDR), developed by the National Cancer Intelligence Network (NCIN) since this project was conducted, is one such dataset that allows each English patient’s treatment pathway to be mapped from diagnosis to cure or death by combining key data from cancer registries and Hospital Episode Statistics (HES). It has been possible to match data collected as part of the Conventional versus Laparoscopic-Assisted Surgery in Colorectal Cancer (CLASICC) clinical trial in colorectal cancer to the NCDR and obtain treatment and outcome data [6]. A similar project has been completed using the TACT breast cancer trial to see if it possible to identify trial patients in the NCDR dataset, to monitor their progress and detect any detrimental effects from their cancer treatment that may have not been previously identified [7]. The capabilities of such a method of LTFUP data collection in Scotland were also assessed for the TACT trial [8]. Results from the TACT projects indicate that data relating to disease recurrence - an essential component of the trials included in this review - are not yet reliably ascertainable via routine sources and that site-based follow-up is still required. More recently, other routine cancer datasets have become available including the Systemic Anti-Cancer Therapy dataset, National Radiotherapy Dataset, and the Cancer Outcomes and Services Dataset (COSD). COSD replaces the previous National Cancer Dataset as the new national standard for reporting cancer in the NHS in England and was implemented in January 2013. These datasets combined with the existing HES data greatly increase the potential offered by the NCIN by including more detail on cancer treatments and reporting of disease relapse.

Meta-analyses of trials continue to offer the most robust estimates of safety and long-term survival outcomes. Breast cancer trialists have been at the forefront of such work via the Oxford based Early Breast Cancer Trialists’ Collaborative Group and it is important that the UK is in a position to continue to contribute to these and other analyses. Using routine datasets to collect LTFUP information on trial patients should offer the greatest potential here; however, validity and reliability of the new datasets are still under evaluation. Once such assurances are in place, use of routinely collected data, which enables trial data to be complemented with population-based data, will enable wider comparison of the characteristics of patients participating in trials with the general population and analysis of other disease and co-morbid outcomes such as incidence of cardiac disease.

## Conclusion

Many of the newer trials on the NIHR breast cancer portfolio are in smaller, more targeted populations and/or with short-term endpoints; thus, fewer patients will - in the future - require LTFUP. However, due to the common incidence of the disease and the recognised scientific importance of questions to be addressed via large pragmatic trials, follow-up still has the potential to be a considerable burden for participating hospitals, CTUs and research funders. Improved communication and data processes between CTUs and participating sites will help manage the workload, whilst improvements in access to routine data sources for CTUs are welcome. Full use of routine datasets for trial-related activities remains an aspiration; however, with the correct systems in place it is hoped that it can be met within the next 5 years.

## Electronic supplementary material

Additional file 1: **Figure A1.** Protocol and case report form (CRF) review data extraction proforma. **Figure A2**. Questionnaire sent to participating sites. **Figure A3a**. Patient identifiers and tracing patients in the long term. Key patient identifiers and percentage of national trials collecting each type. **Figure A3b**. Collection of National Health Service (NHS) number in relation to year the trial opened for recruitment. **Figure A4a**. Frequency of long-term follow-up by type of trial (1-2 years post-randomisation). **Figure A4b**. Frequency of long-term follow-up by type of trial (2-5 years post-randomisation). **Figure A4c**. Frequency of long-term follow-up by type of trial (5-10 years post-randomisation). (PDF 486 KB)

## Abbreviations

AE: adverse event
CHI: Community Health Index
COSD: Cancer Outcomes and Services Dataset
CRF: case report form
CTU: Clinical Trials Unit
EBC: early breast cancer
GP: General Practitioner
HES: Hospital Episode Statistics
LTFUP: long-term follow up
NCDR: National Cancer Data Repository
NCIN: National Cancer Intelligence Network
NCRI: National Cancer Research Institute
NCRN: National Cancer Research Network
NHS: National Health Service
NIHR: National Institute for Health Research
RCT: randomised controlled trial
TACT: Taxotere as Adjuvant Chemotherapy.
