# Supplementary material for: The challenges of long-term follow-up data collection in non-commercial, academically-led breast cancer clinical trials: the UK perspective
Source: Trials. 2014 Sep 27;15:379. doi: 10.1186/1745-6215-15-379 (PMC4192347; doi:10.1186/1745-6215-15-379)
Supplement: Supplementary file 1 — Additional file 1: Figure A1. Protocol and case report form (CRF) review data extraction proforma. Figure A2. Questionnaire sent to participating sites. Figure A3a. Patient identifiers and tracing patients in the long term. Key patient identifiers and percentage of national trials collecting each type. Figure A3b. Collection of National Health Service (NHS) number in relation to year the trial opened for recruitment. Figure A4a. Frequency of long-term follow-up by type of trial (1-2 years post-randomisation). Figure A4b. Frequency of long-term follow-up by type of trial (2-5 years post-randomisation). Figure A4c. Frequency of long-term follow-up by type of trial (5-10 years post-randomisation). (PDF 486 KB) [file 13063_2014_2247_MOESM1_ESM.pdf]

The challenges of long term follow-up data collection in academically-led breast cancer clinical trials: The UK perspective

Lucy S Kilburn, Jane Banerji and Judith M Bliss on behalf of the NCRI Breast Clinical Studies Group

Supplementary material

Figure A1 – Protocol and CRF review data extraction proforma

Name of trial ☐ Trial contact ☐

Type of trial: Chemotherapy ☐ Endocrine therapy ☐ Radiotherapy ☐ Other ☐

Protocol received ☐ Protocol date ☐☐ ☐☐ ☐☐☐☐ (Day, Month, Year)

CRFs received ☐ CRF date ☐☐ ☐☐ ☐☐☐☐ (Day, Month, Year)

Patient information collected at baseline/randomisation:

Patient name	Yes ☐	No ☐
Date of birth	Yes ☐	No ☐
Hospital number	Yes ☐	No ☐
NHS number	Yes ☐	No ☐
Histology number *(if appropriate)*	Yes ☐	No ☐
Location of biological sample *(if appropriate)*	Yes ☐	No ☐

Follow up:

When does follow-up start in trial? ☐

When does follow-up finish in trial? ☐

Which forms are filled in during follow-up, by whom and how often?

What examinations/investigations have to be done during follow-up according to the protocol?

Are in-patient stays recorded? Yes ☐ No ☐

Is there a deviation form? Yes ☐ No ☐ Is there a transfer form? Yes ☐ No ☐

Comments:

Primary endpoint? ☐ **Other recurrence-related endpoints?** Yes ☐ No ☐

Definitions of any recurrence related endpoints according to the protocol

Long term follow-up project template trial information sheet - Version 2 15/10/2008

Name of trial		EFFICACY QUESTIONS

Is efficacy measured?	Yes ☐	No ☐
Is alive/dead status recorded?	Yes ☐	No ☐
If dead, date of death requested?	Yes ☐	No ☐
Patient seen date/phone contact date recorded?	Yes ☐	No ☐

Should the person be followed up in person only, by phone only or can be either?

Person ☐ Phone ☐ Either ☐

Recurrence of breast cancer recorded?	Yes ☐	No ☐	Free text ☐	Tick box ☐	Both ☐
New cancer recorded?	Yes ☐	No ☐	Free text ☐	Tick box ☐	Both ☐

Menstrual status recorded?	Yes ☐	No ☐	How many questions? ☐
Endocrine therapy use recorded?	Yes ☐	No ☐	How many questions? ☐
Concomitant medications post treatment recorded?	Yes ☐	No ☐	How many questions? ☐
QL questions/scoring required?	Yes ☐	No ☐	

Comments

Name of trial		TOXICITY QUESTIONS

Is toxicity measured?	Yes	☐	No	☐
Is there CTC grading via tick boxes?	Yes	☐	No	☐

Are the following toxicities recorded?

Cardiac events	Yes	☐	No	☐	Myalgia/ arthralgia	Yes	☐	No	☐
Neurotoxicity	Yes	☐	No	☐	Fatigue	Yes	☐	No	☐
Hand/foot syndrome	Yes	☐	No	☐	Bone fractures	Yes	☐	No	☐
Hot flushes	Yes	☐	No	☐	Other (free text)	Yes	☐	No	☐

Radiotherapy trials only:

How many side effects are to be assessed? ☐

Is performance status recorded?	Yes	☐	No	☐	
Is weight recorded?	Yes	☐	No	☐	
Any investigations required?	Yes	☐	No	☐	Specify
Any gynaecological procedures done?	Yes	☐	No	☐	

Comments

Name of trial ____ Trial for registration purposes? Yes ☐ No ☐

Sponsor(s) ____ Funder(s) ____

Follow-up:

Will tracing services be used to collect follow-up? Yes ☐ No ☐

If "Yes", specify ____

Visit schedule*	Follow-up form requested?	Who can complete the form?	Investigations to be conducted at visit
3m		Appropriate site personnel ☐ GP ☐ Other (specify) ☐ ____	Blood ☐ ECG ☐ MUGA/ECHO ☐ DEXA ☐ Physical ☐ Photos ☐ Height/weight ☐ Other (specify) ☐ ____
6m		Appropriate site personnel ☐ GP ☐ Other (specify) ☐ ____	Blood ☐ ECG ☐ MUGA/ECHO ☐ DEXA ☐ Physical ☐ Photos ☐ Height/weight ☐ Other (specify) ☐ ____
9m		Appropriate site personnel ☐ GP ☐ Other (specify) ☐ ____	Blood ☐ ECG ☐ MUGA/ECHO ☐ DEXA ☐ Physical ☐ Photos ☐ Height/weight ☐ Other (specify) ☐ ____
12m		Appropriate site personnel ☐ GP ☐ Other (specify) ☐ ____	Blood ☐ ECG ☐ MUGA/ECHO ☐ DEXA ☐ Physical ☐ Photos ☐ Height/weight ☐ Other (specify) ☐ ____
15m		Appropriate site personnel ☐ GP ☐ Other (specify) ☐ ____	Blood ☐ ECG ☐ MUGA/ECHO ☐ DEXA ☐ Physical ☐ Photos ☐ Height/weight ☐ Other (specify) ☐ ____
18m		Appropriate site personnel ☐ GP ☐ Other (specify) ☐ ____	Blood ☐ ECG ☐ MUGA/ECHO ☐ DEXA ☐ Physical ☐ Photos ☐ Height/weight ☐ Other (specify) ☐ ____
24m		Appropriate site personnel ☐ GP ☐ Other (specify) ☐ ____	Blood ☐ ECG ☐ MUGA/ECHO ☐ DEXA ☐ Physical ☐ Photos ☐ Height/weight ☐ Other (specify) ☐ ____
30m		Appropriate site personnel ☐ GP ☐ Other (specify) ☐ ____	Blood ☐ ECG ☐ MUGA/ECHO ☐ DEXA ☐ Physical ☐ Photos ☐ Height/weight ☐ Other (specify) ☐ ____
36m		Appropriate site personnel ☐ GP ☐ Other (specify) ☐ ____	Blood ☐ ECG ☐ MUGA/ECHO ☐ DEXA ☐ Physical ☐ Photos ☐ Height/weight ☐ Other (specify) ☐ ____
42m		Appropriate site personnel ☐ GP ☐ Other (specify) ☐ ____	Blood ☐ ECG ☐ MUGA/ECHO ☐ DEXA ☐ Physical ☐ Photos ☐ Height/weight ☐ Other (specify) ☐ ____
48m		Appropriate site personnel ☐ GP ☐ Other (specify) ☐ ____	Blood ☐ ECG ☐ MUGA/ECHO ☐ DEXA ☐ Physical ☐ Photos ☐ Height/weight ☐ Other (specify) ☐ ____
60m		Appropriate site personnel ☐ GP ☐ Other (specify) ☐ ____	Blood ☐ ECG ☐ MUGA/ECHO ☐ DEXA ☐ Physical ☐ Photos ☐ Height/weight ☐ Other (specify) ☐ ____
72m		Appropriate site personnel ☐ GP ☐ Other (specify) ☐ ____	Blood ☐ ECG ☐ MUGA/ECHO ☐ DEXA ☐ Physical ☐ Photos ☐ Height/weight ☐ Other (specify) ☐ ____
84m		Appropriate site personnel ☐ GP ☐ Other (specify) ☐ ____	Blood ☐ ECG ☐ MUGA/ECHO ☐ DEXA ☐ Physical ☐ Photos ☐ Height/weight ☐ Other (specify) ☐ ____
96m		Appropriate site personnel ☐ GP ☐ Other (specify) ☐ ____	Blood ☐ ECG ☐ MUGA/ECHO ☐ DEXA ☐ Physical ☐ Photos ☐ Height/weight ☐ Other (specify) ☐ ____
108m		Appropriate site personnel ☐ GP ☐ Other (specify) ☐ ____	Blood ☐ ECG ☐ MUGA/ECHO ☐ DEXA ☐ Physical ☐ Photos ☐ Height/weight ☐ Other (specify) ☐ ____
120m		Appropriate site personnel ☐ GP ☐ Other (specify) ☐ ____	Blood ☐ ECG ☐ MUGA/ECHO ☐ DEXA ☐ Physical ☐ Photos ☐ Height/weight ☐ Other (specify) ☐ ____
Other=		Appropriate site personnel ☐ GP ☐ Other (specify) ____	Blood ECG ☐ MUGA/ECHO ☐ DEXA ☐ Physical ☐ Photos ☐ Height/weight ☐ Other (specify) ☐ ____

* time from protocol treatment completion or other (state) ____

Name of trial

Patient information collected at baseline/randomisation:

Gender Yes ☐ No ☐ Ethnicity Yes ☐ No ☐

Mammography:

How often is mammography follow-up requested?

(tick all that apply, e.g. if annual or bi-annual allowed then tick both)

Not requested ☐ Yearly ☐ 2-yearly ☐ 3-yearly ☐

Is the pathology of new primary cancer/contralateral breast cancer collected (if resected)? Yes ☐ No ☐

Are any treatments for relapse/2nd primary collected? Yes ☐ No ☐

Is it via free text or tick box? Free text ☐ Tick box ☐ Both ☐

Radiotherapy trials only:

Is information on the following toxicities/symptoms specifically requested *(e.g. via tick box)*?

Breast shrinkage Yes ☐ No ☐ Lung fibrosis Yes ☐ No ☐

Breast induration Yes ☐ No ☐ Oedema Yes ☐ No ☐

Telangiectasia Yes ☐ No ☐

Comments

Figure A2: Questionnaire sent to centres

Views on long term follow-up data collection for breast cancer clinical trials

This questionnaire is to be completed by <u>any</u> person responsible for completing follow-up forms at your hospital. Please answer all questions in relation to your experiences of <u>academic-led breast cancer clinical trials</u> on the NIHR portfolio only

All responses you provide are treated confidentially and any results presented will not identify you

NCRI National Cancer Research Institute

NCRN National Cancer Research Network

Please return all completed questionnaires to Lucy Kilburn, ICR Clinical Trials & Statistics Unit (ICR-CTSU), The Institute of Cancer Research, Sir Richard Doll Building, Cotswold Road, Sutton, Surrey. SM2 5NG or fax 0208 770 7876

Definition of "follow-up": for the purpose of this questionnaire "follow-up" is defined as visits/contact subsequent to completion of surgery/radiotherapy/chemotherapy treatment

Section 1: General details

Name of your NHS Trust ☐

Name of your hospital ☐

1.1 What is your role in breast cancer trials at your hospital? ***(Please tick one)***

Research Nurse	☐	Consultant Medical/ Clinical Oncologist	☐
Data Manager	☐	Consultant Surgeon	☐
Clinical Trial Assistant	☐	Specialist Registrar	☐
Clinical Trial Officer/Clinical Trial Practitioner/Clinical Research Associate	☐	Other *(specify)*	☐

1.2 Which of the following NIHR portfolio academic-led breast cancer trials is your hospital participating in? ***(Please tick all that apply)***

ABC	☐	ALTTO	☐	ARTEMIS	☐	ATTOM	☐	AZURE	☐
BEATRICE	☐	COMICE	☐	DEVA	☐	EORTC10981	☐	ESTEEM	☐
FAST	☐	HERA	☐	HRT	☐	IMPORT HIGH	☐	IMPORT LOW	☐
LATTE	☐	NEAT	☐	NEO CENT	☐	NEO EXCEL	☐	NEO TANGO	☐
PERSEPHONE	☐	POETIC	☐	PRIME	☐	PRIME II	☐	REACT	☐
SECRAB	☐	SOFT	☐	START	☐	SUPREMO	☐	TACT	☐
TACT2	☐	TANGO	☐	TEAM	☐	TEXT	☐		

Other *(Specify)* ☐

Section 2: General questions about collection of follow-up

2.1 What is the total number of patients who are in the NIHR portfolio trials you have ticked in question 1.2 for whom you are personally responsible for providing follow-up data
(approximately if you do not know exact figure)

2.2 On average how much time each week do you spend completing follow up case report forms for the breast cancer trials listed in question 1.2 conducted in your hospital?

< 2 hours ☐ 2 to 3 hours ☐ 4 to 7 hours ☐ More than 7 hours ☐

Section 3: Questions about follow-up in "routine" practice at your hospital

3.1 How often in "routine" practice are breast cancer patients seen in follow-up clinics after the end of adjuvant treatment (surgery/radiotherapy/chemotherapy)?

	In the first year	In the second year	In the third year	In the fourth year	In the fifth year	In subsequent years
Every 3 months	☐	☐	☐	☐	☐	☐
Every 6 months	☐	☐	☐	☐	☐	☐
Annually	☐	☐	☐	☐	☐	☐
Not seen	☐	☐	☐	☐	☐	☐

Other *(specify)*

3.2 At what point do you discharge patients from follow-up in "routine" practice?

< 3 years after diagnosis ☐ 3-5 years after diagnosis ☐ >5 years after diagnosis ☐ Not discharged ☐

3.3 What investigations are done at each of these visits in "routine" practice?

	Yes, all patients at all visits	Yes, all patients at some visits	Yes, some patients at all visits	Yes, some patients at some visits	No
clinical examination	☐	☐	☐	☐	☐
mammogram	☐	☐	☐	☐	☐
blood test	☐	☐	☐	☐	☐
chest X-ray	☐	☐	☐	☐	☐
ECG	☐	☐	☐	☐	☐

3.4 Do the answers to question 3.1, 3.2 and 3.3 differ according to biology (e.g. ER status), prognosis etc?

Yes, according to biology *(specify)* ☐

Yes, according to prognosis *(specify)* ☐

Yes, according to another factor *(specify)* ☐

No, all patients are treated the same ☐

Section 4: Questions about follow-up practice for patients in breast cancer trials at your hospital

4.1 Which NIHR portfolio trials listed in question 1.2 require you to organise visits in addition to your "routine" practice? *(Please specify name of trial, how many additional visits and when they occur)*

4.2 Which NIHR portfolio trials listed in question 1.2 require you not to discharge patients?

4.3 Which NIHR portfolio trials listed in question 1.2 require you to organise investigations in addition to your "routine" practice? *(Please specify name of trial, how many additional investigations, which investigations and when they occur)*

4.4 Does your hospital run follow-up clinics specifically for trial patients?

Yes ☐ → *Go to Question 4.5*

No ☐ → *Go to Question 4.7*

4.5 If "Yes", are they:

	Nurse-led?	Consultant-led?
General cancer follow-up clinics?	☐	☐
Breast cancer specific follow-up clinics?	☐	☐

4.6 If "Yes", how often are they held?

Weekly ☐

Monthly ☐ *Go to Question 4.9*

6 Monthly ☐

Other *(specify)*

4.7 If "No" has your hospital considered setting up a follow-up clinic for breast cancer trial patients?

Yes ☐ → *Go to Question 4.8*

No ☐ → *Go to Question 4.9*

4.8 If "Yes", why did you decide not to set one up? *(tick all that apply)*

Lack of physical space ☐

Lack of time in outpatient clinic timetable ☐

Lack of time in nurse/consultant timetable ☐

Other *(specify)*

4.9 How do you prioritise follow-up requests coming from different Trial Units?

Complete forms for each request as it arrives ☐

Complete forms for several trials together in batches ☐

Other system *(specify)*

4.10 Do you have a flagging system to identify for which breast cancer trial patients follow-up data is due?

Yes, we proactively follow-up patients before requests are sent from trials units ☐

No, we wait for the trials unit to send out a request ☐

Other method ☐

Please describe your method for flagging these patients

Section 5: Specific questions about collection of follow-up

5.1 How do you obtain follow-up information requested by Trial Units in trial patients *before and after* the patient is discharged? *(tick all that apply)*

	Before patient is discharged	After patient is discharged
The patient's medical record (paper copy)	☐	☐
The electronic Hospital Information System (HIS)	☐	☐
Picture Archiving Computer System (PACS)	☐	☐
Electronic Prescription Service (EPS)	☐	☐
Request patient in for visit	☐	☐
Contact patient's GP	☐	☐
Contact the patient	☐	☐
Other *(specify)*		

5.2 Which of the following items are available on the electronic patient record system your hospital uses?
(tick all that apply)

- SNOMED CT coding ☐
- ICD10 coding ☐
- OPCS4 coding ☐
- Prescriptions ☐
- Dates of relapse ☐
- Dates of second malignancy ☐
- Sites of relapse ☐
- Date of death ☐
- Cause of death ☐
- Hospitalisation details ☐

Toxicity *(specify)*

Other *(specify)*

5.3 Do the patient records you use to annotate follow-up data contain information about late adverse events e.g. fractures/cardiovascular problems?

Yes ☐ No ☐

If "Yes", please provide details

5.4 How easy is it to obtain death certificates from hospital notes or electronic information systems?

Very easy ☐ Fairly easy ☐ Possible, but difficult ☐ Impossible ☐

Describe any difficulties (overleaf if necessary)

5.5 How easy is it to obtain pathology/radiology reports/scans for relapse/second malignancies from hospital notes or electronic information systems?

Very easy ☐ Fairly easy ☐ Possible, but difficult ☐ Impossible ☐

Describe any difficulties (overleaf if necessary)

Section 6: Opinions on follow-up in breast cancer clinical trials

Your opinion and thoughts on how Trial Units currently conduct long term follow up are important and may help us to refine the process for future breast cancer trials.

6.1 In your opinion, what are the difficulties affecting your hospital's ability to provide long term follow-up data?

6.2 Electronic follow-up collection is a possible way forward. In your opinion, which of the issues (e.g. firewalls, password issues, training of staff) are likely to be most problematic?

6.3 Please use this space to comment on long term follow up for breast cancer patients in trials, and what problems if any, (e.g. cost of postage) it presents to your hospital.

6.4 What solutions would help to reduce the resource burden for your hospital?

Please return completed questionnaire to Lucy Kilburn, ICR Clinical Trials & Statistics Unit (ICR-CTSU), The Institute of Cancer Research, Sir Richard Doll Building, Cotswold Road, Sutton, Surrey. SM2 5NG or fax 0208 770 7876.

Thank you for completing this questionnaire

Figure. A3a

Patient identifiers and tracing patients in the long term

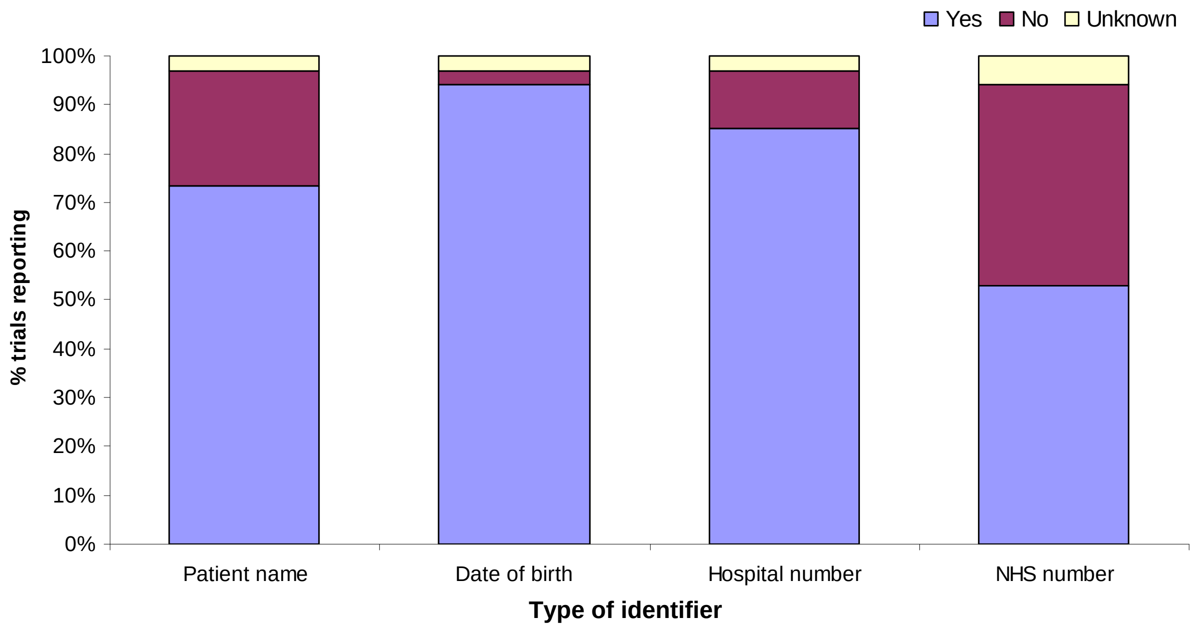

Figure A3a: key patient identifiers and percentage of national trials collecting each type.

Figure A3b.

Collection of NHS number.

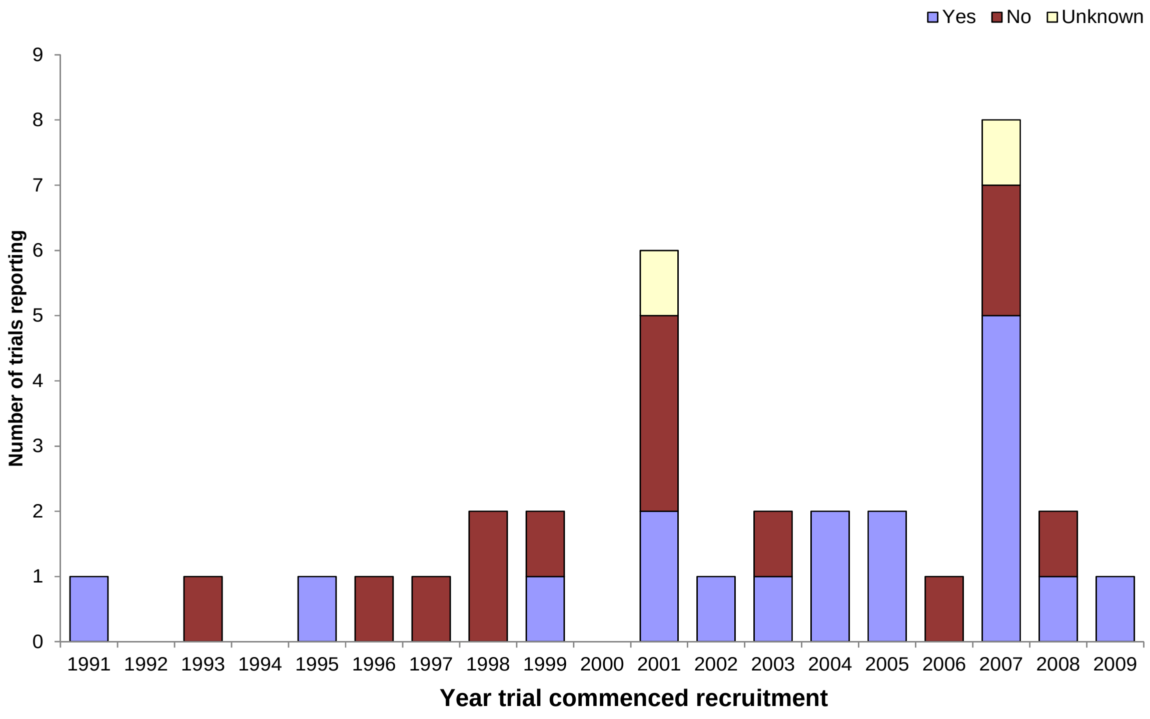

Figure A3b: Collection of NHS number in relation to year the trial opened for recruitment

Figure A4a - Frequency of long term follow-up by type of trial.

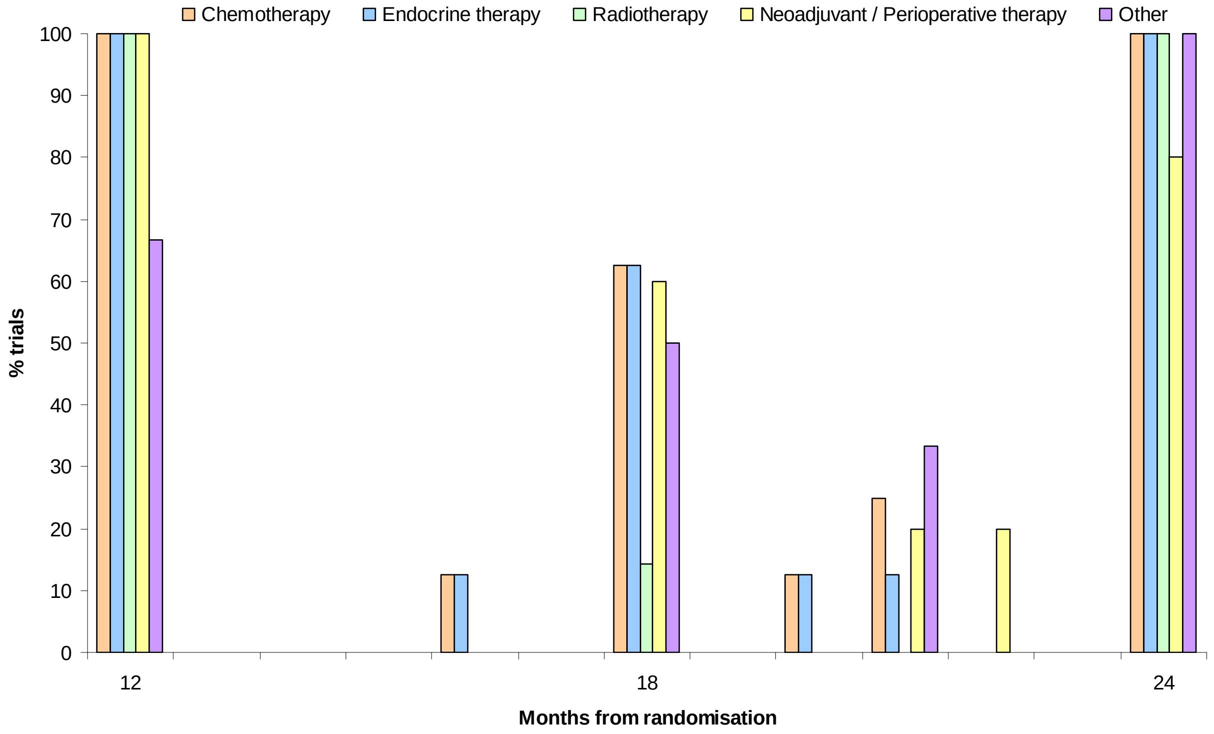

Figure A4a: Frequency of long term follow-up by type of trial - **1-2 years post randomisation**

Figure A4b - Frequency of long term follow-up by type of trial

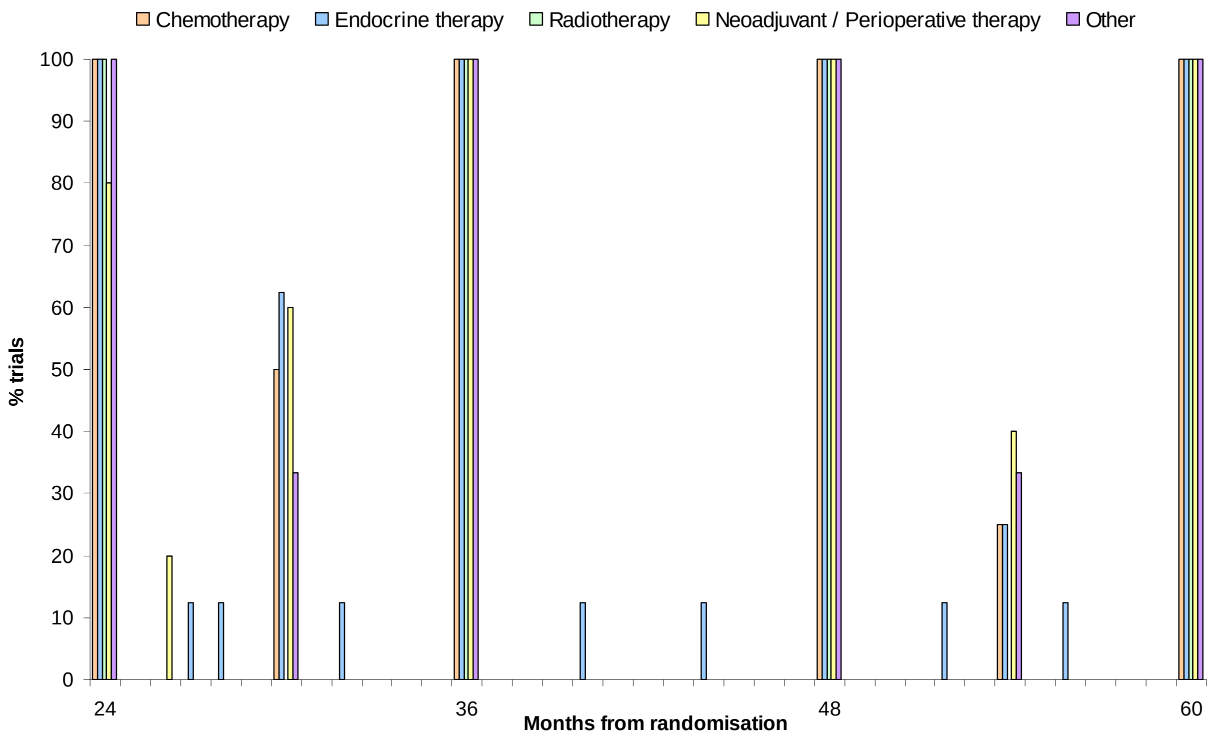

Figure A4b: Frequency of long term follow-up by type of trial - **2-5 years post randomisation**

Figure A4c - Frequency of long term follow-up by type of trial.

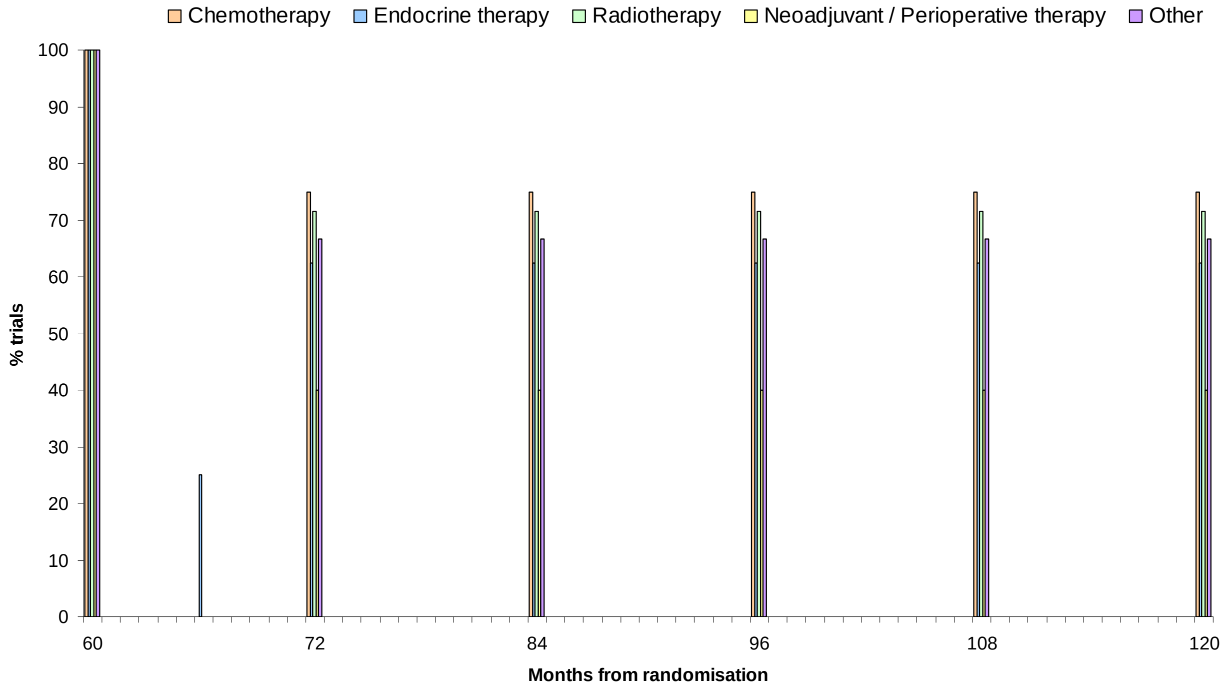

Figure A4c: Frequency of long term follow-up by type of trial - **5-10 years post randomisation.**
